# Converting *F*_ENO_ by different flows to standard flow *F*_ENO_

**DOI:** 10.1111/cpf.12574

**Published:** 2019-06-03

**Authors:** Paul G. Lassmann‐Klee, Lauri Lehtimäki, Tuula Lindholm, Leo Pekka Malmberg, Anssi R.A. Sovijärvi, Päivi Liisa Piirilä

**Affiliations:** ^1^ Unit of Clinical Physiology Helsinki University Central Hospital and University of Helsinki Helsinki Finland; ^2^ Allergy Centre Tampere University Hospital Faculty of Medicine and Health Technology University of Tampere Tampere Finland; ^3^ Department of Clinical Physiology Finnish Institute of Occupational Health Helsinki Finland; ^4^ Laboratory of Clinical Physiology Skin and Allergy Hospital Helsinki University Hospital Helsinki Finland

**Keywords:** adults, alveolitis, asthma, children, COPD, fractional exhaled nitric oxide (*F*_ENO__)_, mouthwash, multiple‐flow

## Abstract

In clinical practice, assessment of expiratory nitric oxide (*F*_ENO_) may reveal eosinophilic airway inflammation in asthmatic and other pulmonary diseases. Currently, measuring of *F*_ENO_ is standardized to exhaled flow level of 50 ml s^−1^, since the expiratory flow rate affects the *F*_ENO_ results. To enable the comparison of *F*_ENO_ measured with different expiratory flows, we firstly aimed to establish a conversion model to estimate *F*_ENO_ at the standard flow level, and secondly, validate it in five external populations. *F*_ENO_ measurements were obtained from 30 volunteers (mixed adult population) at the following multiple expiratory flow rates: 50, 30, 100 and 300 ml s^−1^, after different mouthwash settings, and a conversion model was developed. We tested the conversion model in five populations: healthy adults, healthy children, and patients with COPD, asthma and alveolitis. *F*_ENO_ conversions in the mixed adult population, in healthy adults and in children, showed the lowest deviation between estimated ${\hat{F}}_{\mathrm{ENO}}$ from 100 ml s^−1^ and measured *F*_ENO_ at 50 mL s^−1^: −0·28 ppb, −0·44 ppb and 0·27 ppb, respectively. In patients with COPD, asthma and alveolitis, the deviation was −1·16 ppb, −1·68 ppb and 1·47 ppb, respectively. We proposed a valid model to convert *F*_ENO_ in healthy or mixed populations, as well as in subjects with obstructive pulmonary diseases and found it suitable for converting *F*_ENO_ measured with different expiratory flows to the standard flow in large epidemiological data, but not on individual level. In conclusion, a model to convert *F*_ENO_ from different flows to the standard flow was established and validated.

## Introduction

Chronic bronchial inflammation of the respiratory mucosa can lead to bronchial hyperreactivity and airway obstruction. Clinicians often employ fractional exhaled nitric oxide (*F*_ENO_) to evaluate bronchial eosinophilic inflammation (NICE, 2017). *F*_ENO_ values are flow‐dependent, and an expiratory flow rate of 50 ml s^−1^ mirrors the bronchial nitric oxide (NO) production and not the NO with peripheral origin (Tsoukias & George, 1998; Högman *et al*., 2000). For this reason, *F*_ENO_ measurement is currently standardized at the expiratory flow rate of 50 ml s^−1^ (ATS/ERS, 2005, Horváth *et al*., 2017). Prior to the standardization, *F*_ENO_ was acquired in Northern Europe with expiratory flow rates of 50‐300 ml s^−1^ (Högman *et al*., 1997; Ekroos *et al*., 2002; Rouhos *et al*., 2008) and a previous guideline endorsed the use of flow rates between 167 and 250 ml s^−1^ (Kharitonov *et al*., 1997). Many pioneers in *F*_ENO_ investigation adopted a flow rate of 100 ml s^−1^ (Kharitonov & Barnes, 2001). Unfortunately, data measured at different flow levels have been difficult to compare, since *F*_ENO_ values are affected by the flow rate used and represent NO from anatomically different lung parts. Therefore, a conversion method to interpolate *F*_ENO_ values to equivalent *F*_ENO_ values at diverse flows was needed. Since the lowering effect of mouthwashes on *F*_ENO_ values is well documented (Lassmann‐Klee *et al*., 2018a, 2018b), the conversion method should address also the mouthwashes. The aim of this study was to establish a method for converting *F*_ENO_, measured at different expiratory flow levels, to the standard *F*_ENO_ measured at 50 ml s^−1^ and validate this method. Further on, we aimed to determine the need of considering the mouthwashes in the conversion method.

### Glossary

*F*_ENO_, Fractional exhaled nitric oxide

${\hat{F}}_{\mathrm{ENO}}$, Estimated fractional exhaled nitric oxide

$\dot{V}$, Expiratory flow rate

NO, Nitric Oxide

## Methods

### Data acquisition

We recruited 30 healthy or asthmatic adults as volunteers (henceforth referred as ‘mixed adult population’) to develop a conversion method. We have previously described this population (Lassmann‐Klee *et al*., 2018b). The volunteers were adult patients (*n* = 9) or healthcare workers (*n* = 21). The patients invited were previously referred for *F*_ENO_ assessment to the Laboratory of Clinical Physiology or to the Skin and Allergy Hospital at the Helsinki University Central Hospital area. The healthcare employees were included in the study without exclusions. The patients enrolled had respiratory symptoms or a chronic respiratory disease, including asthma (*n* = 4), eosinophilic bronchitis (*n* = 1), building‐related respiratory symptoms (*n* = 3) and Sjögren's syndrome (*n* = 1). Spirometric data (*n* = 25) were analysed, and none of the participants had actual bronchodilator reversibility (Pellegrino *et al*., 2005).

*F*_ENO_ measurements were performed at the Finnish Institute of Occupational Health and at the Skin and Allergy Hospital with CLD 88 sp chemiluminescence NO analysers and EXHALIZER®'s D devices using SPIROWARE^®^ software (Eco Medics AG, Switzerland). The devices were calibrated in compliance with the producer's specifications: use of certified span gas (AGA Gas BV, Amsterdam, Netherlands) and a zero‐air filtering system (DENOX 88 unit). Additionally, a calibration syringe (Hans Rudolph Inc., USA) was used to calibrate the ultrasonic flow sensor. We complied with all advices from the ATS/ERS statement (ATS/ERS, 2005).

We performed *F*_ENO_ measurements in our mixed adult population (*n* = 30) from September 2016 until May 2017, and the tests for each volunteer were scheduled on 2 consecutive days. All the 30 volunteers followed a mouthwash protocol with tap water and carbonated water. Detailed description of the mouthwashes’ protocol is available in our recent study (Lassmann‐Klee *et al*., 2018b). Briefly, the *F*_ENO_ measurements were performed after a mouthwash with 100 ml of tap water at each flow level. After 15 min, all measurements were repeated after a mouthwash with 100 ml of carbonated water at each flow level. The mouthwashes’ effect, duration and chemical composition are well documented (Lassmann‐Klee *et al*., 2018a, 2018b).

Secondly, we selected 10 healthcare workers from the aforementioned volunteers to perform an additional measurement phase. The selection criterion was inclusion only of those employed at the Skin and Allergy Hospital. In the third appointments, the 10 healthcare workers performed the measurements without a mouthwash.

*F*_ENO_ was acquired from all participants at the following multiple expiratory flow rates: 50, 30, 100 and 300 ml s^−1^. At least two measurements of *F*_ENO_ were obtained at each flow level. The values were accepted, if its variation was less than 2 ppb.

### Validation

For validating our conversion method, 5 different datasets of previously published articles acquired at the Tampere University Hospital were available. They contained multiple‐flow data from 69 healthy adults (Lehtimäki *et al*., 2010a, 2010b), 66 healthy children (Sepponen *et al*., 2008), 74 steroid‐naive adults with COPD (Lehtimäki *et al*., 2010a), 40 steroid‐naive adults with asthma (Lehtimäki *et al*., 2001) and 17 subjects with untreated alveolitis (Lehtimäki *et al*., 2001). The validation process is explained in the statistical section.

This study followed the ethical principles of the declaration of Helsinki (World Medical Association Declaration of Helsinki, 2013) and received approval from an ethical committee (99/13/03/00/15). All participants signed an informed consent.

### Statistics

#### Modelling the conversion method

Analyses were performed using RSTUDIO® version 1·1·383 frontend to the R statistics language (R Core Team, 2018). We agreed on a significance level of α = 0·05 as significant. We calculated the arithmetic mean from individual *F*_ENO_ values obtained at each flow level. The mean values were plotted against the expiratory flow rate $\dot{V}$ in a double logarithmic scale, and we performed a non‐linear regression. We obtained a slope and intercept and analysed the regression line to develop our conversion model. To further refine the model, we acquired a non‐linear least squares estimation of the non‐linear model parameters. This model was used to estimate ${\hat{F}}_{\mathrm{ENO}}$ values from *F*_ENO_ values measured at different flow rates.

### Validation

To test the validity of our model, we converted *F*_ENO_ values measured at 30, 100 and 300 ml s^−1^ to estimated ${\hat{F}}_{\mathrm{ENO}}$ values for a standard flow rate of 50 ml s^−1^. Afterwards, we compared the estimated ${\hat{F}}_{\mathrm{ENO}}$ values to the actual *F*_ENO_ measured at 50 ml s^−1^. To assess the agreement between estimated ${\hat{F}}_{\mathrm{ENO}}$ and measured *F*_ENO_, we performed an analysis (see below) according to Bland & Altman (2010). Further on, the correlation coefficient rho was obtained with Spearman's formula to investigate linearity.

To validate our conversion model in different external populations, we compared the estimated ${\hat{F}}_{\mathrm{ENO}}$ converted from 100 ml s^−1^ with *F*_ENO_ measured at 50 or 40 ml s^−1^. For this external validation, a method described by Bland & Altman (2010) was employed. Accordingly, we obtained the individual differences of *F*_ENO_, the mean of differences (bias) and the 1·96 standard deviations of the mean (95% limits of agreement).

Additionally, we performed a linear regression analysis (glm) between *F*_ENO_ values measured at 50 ml s^−1^ after the tap water and carbonated water mouthwashes, to obtain a relation between the mouthwashes and to provide an additional equation to convert measurements with these two mouthwashes to the standard flow level (50 ml s^−1^).

When necessary, raw data were examined for outliers using the absolute deviation around the median (3 deviations as threshold). If cases were omitted, the conversion was repeated and the differences and level of agreements adjusted (Leys *et al*., 2013).

## Results

### Conversion model

We plotted the mean *F*_ENO_ values against the expiratory flow rate $\dot{V}$ and performed a non‐linear regression. Acquiring non‐linear least squares parameter estimates resulted in a slope of −0·8416 SE(0·3192) for carbonated water, a slope of −0·84 SE(0·2989) for tap water and a slope of −0·83111 SE(0·05424) in the absence of a mouthwash. In the latter case, the equation model can be further defined as:

(1) $${\hat{F}}_{\mathrm{ENO}}=k\cdot {\dot{V}}^{-0\cdot 83111}$$

**Figure 1:**
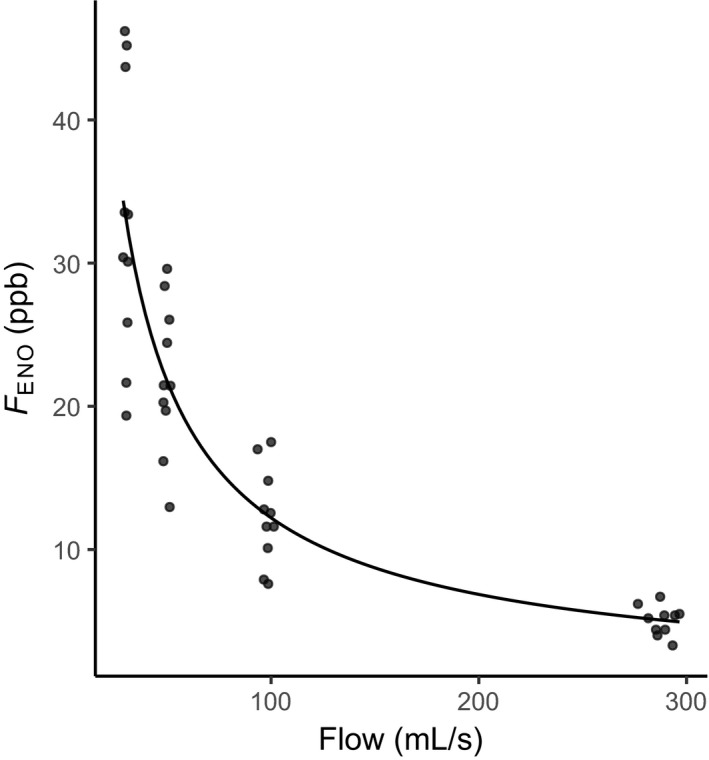
*F*_ENO_ as a function of expiratory flow (without mouthwash), *n* = 10. Curve shows the equation${\hat{F}}_{\mathrm{ENO}}=k\cdot {\dot{V}}^{-0\cdot 83111}$.

Plotting our model with Eq.  using measured *F*_ENO_ and $\dot{V}$, as well as calculated values for *k*, resulted in Fig. 1.

The linear regression of *F*_ENO_ at 50 ml s^−1^ after a tap water mouthwash in relation to carbonated water resulted in a slope coefficient of 1·055 ppb and intercept of 0·354 ppb (*P*<0·001).

When employing the different estimating slopes for the ${\hat{F}}_{\mathrm{ENO}}$ conversions with tap water and carbonated water mouthwashes, the mean estimated ${\hat{F}}_{\mathrm{ENO}}$ for the carbonated water mouthwash was ca. −4·5% lower than the mean estimated ${\hat{F}}_{\mathrm{ENO}}$ for tap water at all flow levels (unadjusted).

### Validation results in mixed adult population

Using Eq. (1), we calculated the values for ${\hat{F}}_{\mathrm{ENO}}$ (flow level 50 ml s^−1^) interpolated from data obtained at 100 ml s^−1^. Applying the (Bland & Altman, 2010) method resulted in mean (SD) differences between the estimated ${\hat{F}}_{\mathrm{ENO}}$ (flow level 50 ml s^−1^) and the measured *F*_ENO_ (flow level 50 ml s^−1^) of ‐0·45(2·44) ppb, upper 95% limit of agreement of 4·34 ppb and lower 95% limit of agreement of −5·23 ppb. The measured *F*_ENO_ and the estimated ${\hat{F}}_{\mathrm{ENO}}$ had a good correlation (Spearman's ρ = 0·87; *P*<0·0001).

**Figure 2:**
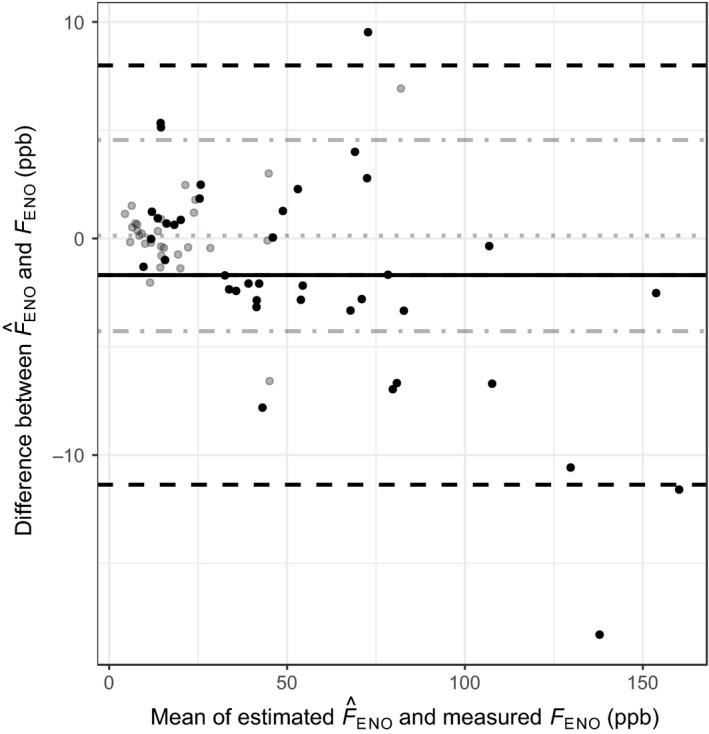
Bland–Altman plot with mean of measured *F*_ENO_ and estimated ${\hat{F}}_{\mathrm{ENO}}$ from 100 ml s^−1^ in asthmatics (grey dots, *n* = 40) and our mixed adult population (black dots, *n* = 30), plotted against the differences in *F*_ENO_. In asthmatics: mean differences (grey dotted line), 1·96 standard deviations (grey dot‐slashed line). In mixed adult population: mean differences (black solid line), 1·96 standard deviation (black slashed line). In asthmatics *F*_ENO_ measured at 40 ml s^−1^. In mixed adult population *F*_ENO_ measured at 50 ml s^−1^ after carbonated water mouthwash.

We also estimated ${\hat{F}}_{\mathrm{ENO}}$ (50 ml s^−1^) from values measured at all flow levels and mouthwash settings. All differences with the (Bland & Altman, 2010) method showed a good agreement, and the total unadjusted mean of the absolute deviation of ${\hat{F}}_{\mathrm{ENO}}$ from *F*_ENO_ was 0·72 ppb. All estimated values were highly correlated with corresponding measured values. Table 1 summarizes these results. Figure 2 exemplifies the unadjusted mean differences of ${\hat{F}}_{\mathrm{ENO}}$ and *F*_ENO_ after applying Eq. (1) (conversion with carbonated water mouthwash from flow of 100 ml s^−1^). After adjusting measured *F*_ENO_ by removing outliers and performing a new estimation, a better agreement was found between estimated ${\hat{F}}_{\mathrm{ENO}}$ and measured *F*_ENO_, and total mean of the absolute deviations of ${\hat{F}}_{\mathrm{ENO}}$ from *F*_ENO_ was 0·66 ppb. The adjusted results after controlling for outliers can be also found in Table 1.

**Table 1:** Bland–Altman statistics in our mixed healthy and asthmatic adult population (*n* = 30) and in healthcare workers (*n* = 10) with mean, bias^a^, levels of agreement and standard deviation (SD) of the differences between estimated ${\hat{F}}_{\mathrm{ENO}}$ from different flow levels and mouthwashes, and measured *F*_ENO_ at 50 ml s^−1^ (tap water: 27·27 ppb; carbonated water: 25·51 ppb; no mouthwash: 22·05)

Mean estimated ${\hat{F}}_{\mathrm{ENO}}$ (ppb) at 50 ml s^−1^ from flow level and mouthwash		Bias^a^				Adjusted values					
			Level of agreement			Level of agreement					
			Lower	Upper	SD	bias^a^	Lower	Upper	SD	rho	b
30 ml s^−1^; tap	25·24	−2·03	−11·17	7·10	4·66	−1·23	−5·44	3·0	2·15	0·96	3
100 ml s^−1^; tap	26·99	−0·28	−7·42	6·86	3·64	−0·11	−3·67	3·44	1·81	0·98	3
300 ml s^−1^; tap	26·27	−1·00	−19·02	17·01	9·19	0·74	−5·79	7·27	3·33	0·95	2
30 ml s^−1^; carbonated	24·23	−1·28	−4·92	2·36	1·86	−1·50	−4·90	1·90	1·73	0·99	3
100 ml s^−1^; carbonated	25·65	0·13	−4·28	4·55	2·25	−0·08	−3·32	3·16	1·65	0·99	4
300 ml s^−1^; carbonated	25·07	−0·44	−13·32	12·43	6·57	0·99	−4·69	6·67	2·90	0·95	4
30 ml s^−1^; no mouthwash	21·64	−0·41	−5·89	5·06	2·79	−0·41	−5·89	5·06	2·79	0·84	0
100 ml s^−1^; no mouthwash	21·60	−0·45	−5·23	4·34	2·44	−0·45	−5·23	4·34	2·44	0·87	0
300 ml s^−1^; no mouthwash	21·62	−0·43	−5·67	4·82	2·68	−0·43	−5·67	4·82	2·68	0·82	0

Raw data and adjusted values for outliers. Rho according to Spearman's test.

a average of the differences between estimated ${\hat{F}}_{\mathrm{ENO}}$ and measured *F*_ENO_.

b Number of observations excluded with the adjustment.

### Validation results in external populations

**Figure 3:**
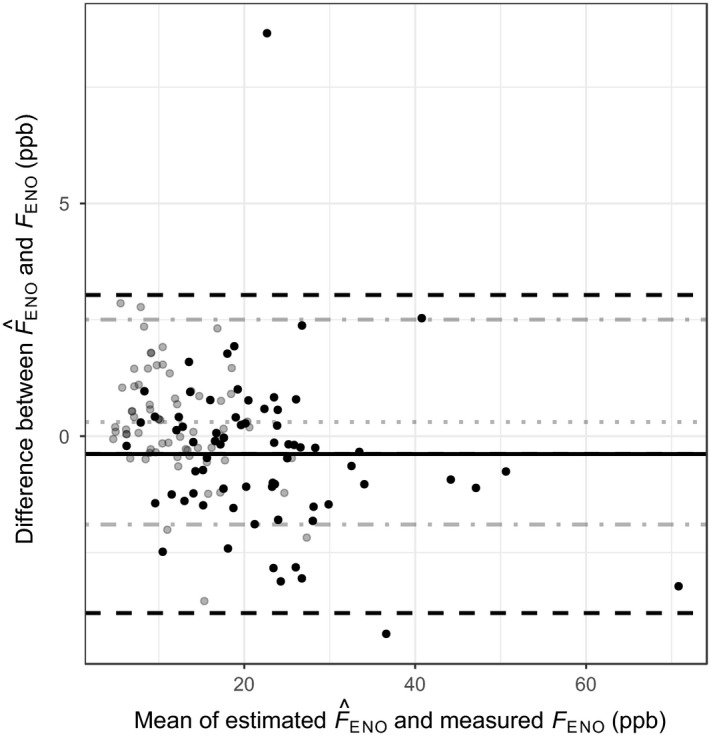
Bland–Altman plot with mean of *F*_ENO_ measured at 50 ml s^−1^ and estimated ${\hat{F}}_{\mathrm{ENO}}$ from 100 ml s^−1^ in healthy children (grey dots, *n* = 66) and in healthy adults (black dots, *n* = 69), plotted against the differences in *F*_ENO_. In healthy children: mean differences (grey dotted line), 1·96 standard deviations (grey dot‐slashed line). In healthy adults: mean differences (black solid line), 1·96 standard deviation (black slashed line).

**Figure 4:**
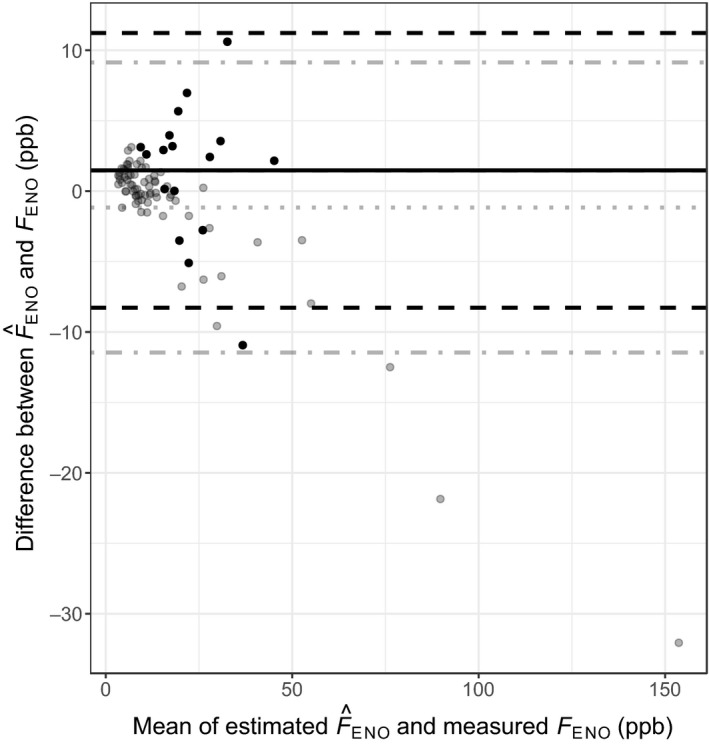
Bland–Altman plot with mean of measured *F*_ENO_ and estimated ${\hat{F}}_{\mathrm{ENO}}$ from 100 ml s^−1^ in COPD patients (grey dots, *n* = 72) and patients with alveolitis (black dots, *n* = 17), plotted against the differences in *F*_ENO_. In COPD patients: mean differences (grey dotted line), 1·96 standard deviations (grey dot‐slashed line). In patients with alveolitis: mean differences (black solid line), 1·96 standard deviation (black slashed line). In patients with alveolitis *F*_ENO_ measured at 40 ml s^−1^. In COPD patients *F*_ENO_ measured at 50 ml s^−1^.

**Figure 5:**
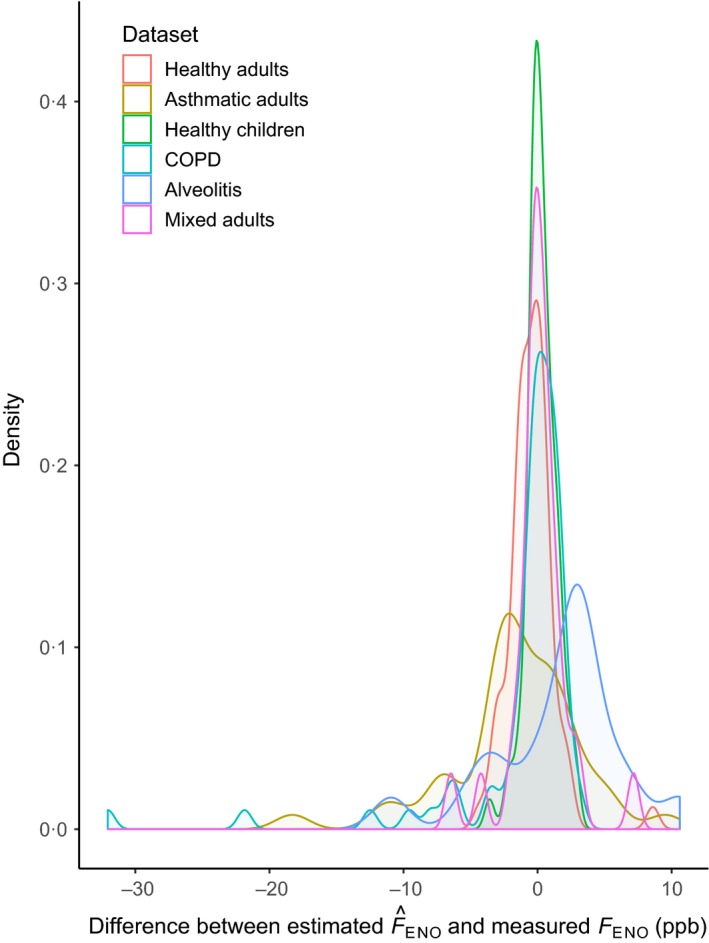
Density plot with mean differences between *F*_ENO_ measured at 50 or 40 ml s^−1^ and estimated ${\hat{F}}_{\mathrm{ENO}}$ from 100 ml s^−1^, and the density of the individual mean differences in all study groups. [Colour figure can be viewed at http://wileyonlinelibrary.com]

With the same approach, we converted *F*_ENO_ data obtained at 100 ml s^−1^ (Lauri Lehtimäki *et al*., 2001; Sepponen *et al*., 2008; Lehtimäki *et al*., 2010a, 2010b) to estimated ${\hat{F}}_{\mathrm{ENO}}$ (flow level 50 or 40 ml s^−1^) without a mouthwash (Eq. ). The mean difference between estimated ${\hat{F}}_{\mathrm{ENO}}$ and measured *F*_ENO_ was lowest (0·27 ppb) in the healthy children group, followed by the healthy adult group (−0·44 ppb), as shown in Fig. 3. The mean difference illustrated in Fig. 2 of steroid‐naive adults with asthma was −1·68 ppb. In Fig. 4, the mean difference shown is −1·16 ppb in steroid‐naive adults with COPD, and 1·47 in the untreated alveolitis population. The healthy groups had narrow limits of agreement, in contrast to the groups with diseases. Table 2 synthesizes these results. Additionally, Fig. 5 demonstrates the distribution of the differences in all populations. Table 3 contains the correlation between the measured and estimated *F*_ENO_ values and provides information concerning the linearity between the values.

**Table 2:** Bland–Altman statistics with bias^a^, levels of agreement and standard deviation (SD) of the differences between estimated ${\hat{F}}_{\mathrm{ENO}}$ from 100 ml s^−1^ (Eq. 1) and measured *F*_ENO_ at 50 or 40 ml s^−1^

Population	Bias^a^	Level of agreement		SD
		Lower	Upper	
Mixed healthy and asthmatic adults	−0·28	−7·42	6·86	3·64
Healthy adults	−0·44	−3·87	2·98	1·74
Asthmatic	−1·68	−11·36	7·99	4·94
Healthy children	0·27	−1·94	2·48	1·13
COPD	−1·16	−11·46	9·13	5·25
Alveolitis	1·47	−8·28	11·22	4·98

a average of the differences between estimated ${\hat{F}}_{\mathrm{ENO}}$ and measured *F*_ENO§._

**Table 3:** Spearman's correlation between estimated ${\hat{F}}_{\mathrm{ENO}}$ from 100 ml s^−1^ and measured *F*_ENO_ at 50 ml s^−1^, with 95% CI and *P* values

Population	Correlation	95% CI		*P*
		Lower	Upper	
Mixed healthy and asthmatic adults	0·99	0·98	0·99	<0·001
Healthy adults	0·97	0·95	0·98	<0·001
Asthmatic	0·99	0·98	0·99	<0·001
Healthy children	0·97	0·95	0·98	<0·001
COPD	0·98	0·96	0·98	<0·001
Alveolitis	0·87	0·68	0·95	<0·001

## Discussion

### Conversion model

We found that using a non‐linear regression yielded a simple model to convert *F*_ENO_ values measured at different flows to estimated ${\hat{F}}_{\mathrm{ENO}}$ at 50 ml s^−1^. To prove the feasibility of the equation, we compared estimated ${\hat{F}}_{\mathrm{ENO}}$ levels at the standard flow (50 ml s^−1^) from all flow levels (30, 100 and 300 ml s^−1^), with *F*_ENO_ acquired at 50 ml s^−1^ and found a good mean agreement between the estimated and measured values. The limits of agreement between estimated ${\hat{F}}_{\mathrm{ENO}}$ and *F*_ENO_ were reasonable.

### Validation

Assessment of the conversion in external datasets, including data of a wide range of pulmonary diseases and multiple‐flow *F*_ENO_ values, confirmed these previous findings. The conversion model developed showed the lowest deviation in *F*_ENO_ conversions in healthy children, healthy adults and in our mixed asthmatic and healthy adult population. In the steroid‐naive asthmatic, alveolitis and COPD populations, the average differences in *F*_ENO_ were moderate with moderate limits of agreement. In the population with COPD, some single individuals showed a considerable deviation.

We acknowledge the limitation of this conversion procedure, that is being only an approximation that may result in a considerable deviation between estimated and physiological values especially at extreme *F*_ENO_ and/or flow levels, as observed in conversions from low flow (30 ml s^−1^) or high expiratory flow (300 ml s^−1^) levels. Nevertheless, this equation is useful when comparing the *F*_ENO_ medians of large population data measured at different flow levels, being very reliable on the group level, although not on individual level. The conversion model developed suits best *F*_ENO_ conversions in healthy adults, healthy children and in a mixed adult population, showing the lowest deviation. This novel conversion model mimics physiological expiratory NO values proportional to expiratory flows. Similar *F*_ENO_ and expiratory flow curves were previously described by other researchers (Tsoukias & George, 1998; Silkoff *et al*., 2000), but this model uses a simplified approach in estimating ${\hat{F}}_{\mathrm{ENO}}$ and makes no claim in predicting flow‐independent parameters.

Since the conversion model developed derives from healthy and asthmatic adults without alveolar diseases, the slope reflects only very low amounts of alveolar nitric oxide concentration (C_ANO_). We previously determined C_ANO_ in our mixed healthy and asthmatic group and all results were under 2·3 ppb (Lassmann‐Klee *et al*., 2018b). Logically, the slope and the estimating equation would change, if switching the participants with subjects with high alveolar NO. The conversion method produces errors in those subjects in whom the relation between alveolar and bronchial NO production is very different from the group mean, as the slope between *F*_ENO_ and $\dot{V}$ is very different in these subjects. Therefore, the model may result in erroneous estimates when applied to subjects with known high alveolar nitric oxide concentrations. Emphasis should be made, not to employ the model without discretion in this type of subjects. The elimination of outliers could represent a limitation of our study, although we did not observe drastic changes when comparing the bias between crude and adjusted data. This statistical adjustment merely narrowed the limits of agreement and served the purpose of demonstrating how the model estimates *F*_ENO_ values stemming from adjusted datasets.

Further on, regression estimates were obtained for *F*_ENO_ values between the mouthwashes, in order to facilitate an interpolation between *F*_ENO_ values measured at 50 ml s^−1^ after carbonated, and tap water, and vice versa. Our estimating equation provides different slopes for both mouthwashes. The mean estimated ${\hat{F}}_{\mathrm{ENO}}$ values were ca. 4% lower for the carbonated water mouthwash than the tap water mouthwash. This approximate difference between these mouthwashes was previously confirmed (Lassmann‐Klee *et al*., 2018a, 2018b). The conversion model succeeds also in considering the mouthwashes.

In conclusion, we developed an equation for converting *F*_ENO_ values obtained with different flow levels to *F*_ENO_ with standard flow (50 ml s^−1^), taking also into account the eventual mouthwash. We proposed a novel model to convert *F*_ENO_ in healthy populations, as well in subjects with obstructive pulmonary diseases. We conclude that the model is reliable in converting *F*_ENO_ in large epidemiological data and might be applied in small scale populations with pulmonary diseases, but not on individual level.

## Funding

This work was supported by the Nordic Council of Ministers, NordForsk Institution (The Nordic EpilLung Study), the Nummela Sanatorium Foundation (PP 2015, 2017), (AS 2016), Finnish State Funding for University‐level Health Research (TYH: 2013354), The Research Foundation of the Pulmonary Diseases (PLK 2017, 2018, 2019), Tampere Tuberculosis Foundation: Eero Hämäläinen (PLK 2017, 2018), Ida Montin Foundation (PLK 2017, 2019), Väinö and Laina Kivi Foundation (PLK 2017, 2018, 2019), and University of Helsinki (PLK 2019).

## Disclosures

No conflicts of interest are declared by the author(s).
